# The effect of risk at birth on breastfeeding duration and exclusivity: A cohort study at a Brazilian referral center for high-risk neonates and infants

**DOI:** 10.1371/journal.pone.0255190

**Published:** 2021-08-06

**Authors:** Maíra Domingues Bernardes Silva, Raquel de Vasconcellos Carvalhaes de Oliveira, Davi da Silveira Barroso Alves, Enirtes Caetano Prates Melo

**Affiliations:** 1 Human Milk Bank at the National Institute of Women, Children and Adolescents Health Fernandes Figueira (IFF) of the Oswaldo Cruz Foundation (FIOCRUZ), Rio de Janeiro, RJ, Brazil; 2 National Institute of Infectious Diseases (FIOCRUZ), Rio de Janeiro, RJ, Brazil; 3 Federal University of the State of Rio de Janeiro (UNIRIO), Rio de Janeiro, RJ, Brazil; 4 National School of Public Health (FIOCRUZ), Rio de Janeiro, RJ, Brazil; Kobe University Graduate School of Medicine, JAPAN

## Abstract

**Background and aim:**

Both breastfeeding and the use of human milk are strategies that provide better conformation to health throughout an individual’s life and bring countless short- and long- term benefits, which are well established in the scientific literature. For at-risk newborns (NBs), these strategies are crucial interventions to enable neonatal survival with better quality of life due to the distinctive and complex composition of human milk, which serves as personalized food-medicine-protection. However, there is limited knowledge about breastfeeding practices in high-risk NBs. The aim was to estimate the duration of EBF and to investigate the effect of risk at birth on EBF discontinuity in the first six months of life’.

**Methods:**

This cohort study included 1,003 NBs from a high-risk referral center, followed up from birth to the sixth month of life, between 2017 and 2018. Correspondence and cluster analysis was used to identify neonatal risk clusters as the main exposure. The object of interest was the time until EBF discontinuity. The Kaplan-Meier methods and the Cox proportional hazards model were used to estimate the hazard ratio and 95% confidence intervals.

**Results:**

The prevalence and median duration of EBF decreased proportionally in the three groups. The multiple model revealed a gradient in EBF discontinuity, which was 40% higher in risk group 1 and 111% higher in risk group 2 compared to healthy full-term NBs. Additionally, EBF during hospitalization predicted a longer median duration of this practice for high-risk NBs.

**Conclusion:**

This study confirms a high proportion of high-risk NBs who have EBF discontinued before six months of life. The risk of EBF discontinuity is higher in risk groups, with a gradual effect even when adjusted by several factors. Effective interventions are needed to promote, protect, and support breastfeeding in different profiles of risk-at-birth groups.

## Introduction

Several benefits of breastfeeding (BF) are well-established in the literature [1–9]. For high-risk newborns (NBs), they range from protection against some diseases to neonatal survival with a higher quality of life [10–20]. Considering that it is an intervention of great impact in the short, medium, and long term, exclusive breastfeeding (EBF) in the first six months and continued until two years of age is strongly recommended for every child [5, 16, 21], and its benefits extend to better health as a whole.

Despite the available evidence, EBF rates and duration remain below recommended levels, especially in high-risk NBs [22–26]. Most countries have low or regular EBF rates for children under six months. The latest Brazilian survey showed an EBF prevalence of 45%, similar to the mean worldwide prevalence in 2018 [27–29]. Diverse behaviors are observed in underdeveloped countries with high BF prevalence at all ages, and BF rate and duration decreases as national wealth increases (except for early initiation) [5].

The definitions of risks for NBs include the characteristics of the mother, the child, and the social and economic context, without the necessary distinction between patterns and types of risk. Few authors have investigated BF in high-risk NBs [30].

Based on this discussion, the aim of this study was to estimate the median duration of EBF and to investigate the effect of risk at birth on EBF discontinuation in the first six months of life in a neonatal cohort from a reference center for high fetal, neonatal, and infant risks in Brazil.

## Materials and methods

### Study design and settings

This was a prospective and dynamic cohort study that included 1,003 NBs born or transferred to the Fernandes Figueira National Institute of Woman, Child, and Adolescent Health of the Oswaldo Cruz Foundation (IFF/FIOCRUZ) up to seven days old. The hospital is a public referral center for high fetal, neonatal, and infant risk, located in Rio de Janeiro, RJ, Brazil and is accredited as the Baby-Friendly Hospital Initiative (BFHI). The IFF/FIOCRUZ is equipped with a Human Milk Bank (HMB), and it is a National Referral Center for the Brazilian Network of Human Milk Banks and a Global Referral Center for 23 cooperating countries. The children were recruited from March 13, 2017 to April 12, 2018, being followed up in their first six months of life. Details on the participants, location, and procedures have been described in another study [31].

### Study population and data collection

Data collection involved three stages, (i) at the maternity hospital, with face-to-face interviews with mothers, and data extraction from hospital records to obtain sociodemographic characteristics and data related to the mother, child, health service and breastfeeding practice; (ii) at the first consultation after hospital discharge at the follow-up clinic or HMB; (iii) monthly telephone interviews until the sixth month of life (up to ten attempts were made per month to minimize follow-up losses). Quality control and assurance measures were established at all data collection stages, such as training and recertification of the team was carried out; pilot studies; pretesting of the system of data entry (use of a web application developed specifically for this research); meetings with the data collection team (at least on a monthly basis and whenever necessary); register of occurrences on the field diary.

### Data measures

To explore the risk patterns for NBs, a multiple correspondence analysis (MCA) was performed with ten characteristics of the selected NBs and mothers based on the national and international definition criteria of the American Academy of Pediatrics and the Brazilian Ministry of Health [32–34], which are similar. According to the Ministry of Health in Brazil [32, 34], the term “newborn at risk” refers to someone exposed to situations in which there is a greater risk of unfavorable evolution. They suggest the following criteria to identify at-risk newborns: low socioeconomic level; a history of death of children under 5 years of age in the family; explicitly unwanted child; adolescent motherhood (< 20 years); preterm newborn (< 37 weeks); infant with low birth weight (< 2,500 g) and mother with low education (< 8 years of schooling). They are also used to identify high-risk newborns: newborns with severe asphyxia at birth (Apgar < 7 in the 5th min); preterm neonates with a birth weight of less than 2,000 g; newborns with less than 35 weeks of gestational age; or newborns with other serious diseases. The American Academy of Pediatrics recognizes the following as high-risk newborns: premature infants (newborns younger than 37 weeks), newborns with special health or technology-dependent needs (children who require some technological support, or nutritional support and respiratory support, including supplemental oxygen), newborns at risk due to family issues (low educational level, lack of social support, marital instability and few visits to prenatal care), and mothers or newborns with premature death (newborns without life expectancy) [33].

Although the definitions make no distinction as to the nature of the risk, aspects related to biological, social, and potential risk are considered. The potential risk refers to the possibility of a health problem, without necessarily describing the disease and its probability of occurrence [35]. The characteristics studied included biological, social, and potential conditions such as birth weight (< 1,500 g, 1,500–2,500 g, and > 2,500 g), gestational age (< 37 and ≥ 37 weeks), genetic syndrome, perinatal morbidity (morbidity at birth, including surgical morbidity and signs that define genetic syndromes), surgical morbidity, fifth minute Apgar score (< 7 or ≥ 7), twinning (twins/triplets/quadruplets), maternal education (up to elementary school, high school, or more), maternal age (< 20, 20–34, and ≥ 35 years), and gestational morbidity.

After the first MCA, the variables that presented a relative contribution to the inertia of the first dimension greater than or equal to 1/number of variables were selected [36]. Then, a second MCA was implemented using the remaining variables. The variables that showed no relative contribution to the inertia were included as supplementary variables. The graphical presentation represents the multivariate distribution of contributions by category on a two-dimensional map, in which the proximity of the points shows a specific data subset (group). To corroborate the correspondence results, the values obtained from the subjects’ main coordinates were included in a cluster analysis partitioned by the Partition Around Medoids algorithm [37].

Considering the cluster contributions, the subjects were grouped according to a variable that identifies the risk group. Associations of mother and NB variables (related to biological, social, and potential risk) according to the groups identified in cluster analysis were verified by the Pearson’s chi-square or Fisher’s exact tests, if the expected frequency was less than 5.

The three NB groups identified by MCA and confirmed by cluster analysis were considered in the exposure analysis. The outcome of interest was defined as the time until EBF discontinuation in the first six months of the child’s life. EBF was defined as an exclusive supply of breast milk, with no other liquids or foods, except medications and vitamins. Children who received liquids such as water, teas and juices, supplements with other types of milk such as infant formula, solid and semi-solid foods, and who were no longer being breastfed were classified as EBF discontinuation [38].

### Data analysis

Absolute and relative frequencies were used for exploratory analysis of qualitative variables. In addition, confidence intervals were provided (CI 95%) to analyze the distribution of qualitative variables by outcome and risk groups.

To verify EBF duration and the effect of risk at birth on EBF discontinuation, a causal diagram represented by the Directed Acyclic Graph (DAG) identified the minimum set of covariables to be included in the multiple Cox regression (See S1 File). It was hypothesized that exposure to different risk levels may proportionally affect EBF in the first six months of life. The DAGitty^®^ browser version 3.0 was used to build the DAG [39].

**Fig 1:**
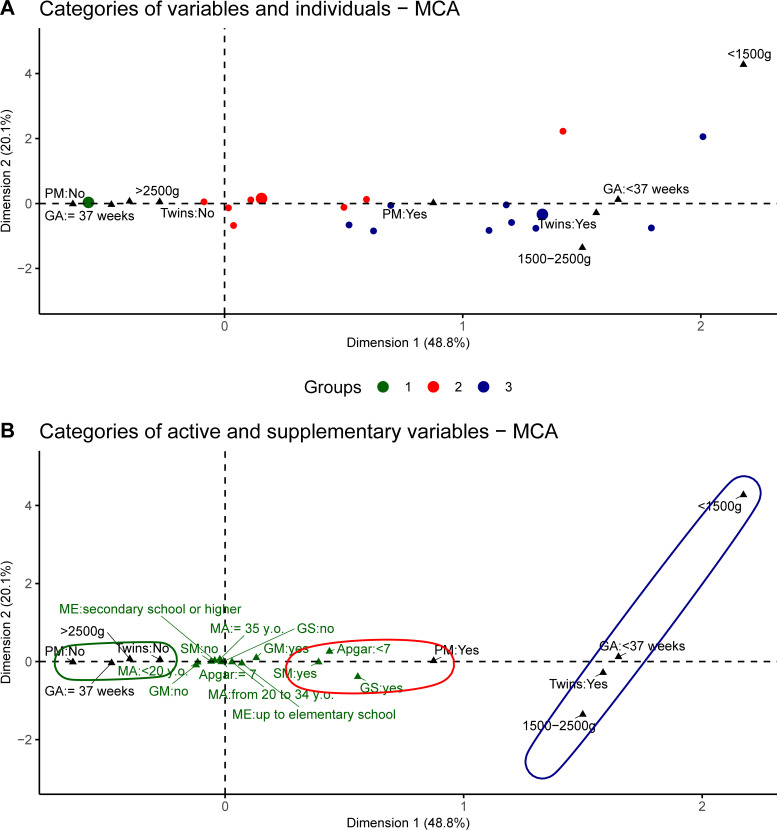
Correspondence analysis map of 1,003 newborns from a high-risk referral center, Rio de Janeiro, RJ, Brazil, 2018. Note: Neonatal grouping: (i) group of healthy full-term newborns; (ii) risk group 1—characterized by NBs with surgical morbidities and genetic syndrome; (iii) risk group 2—characterized by preterm and low birth weight NBs. PM = perinatal morbidity; SM = surgical morbidity; GM = gestational morbidity; GA = gestational age; GS = genetic syndrome; ME = maternal education; MA = maternal age. Log-rank < 0.001.

The confounding variables that represent the total effect of the relationship between high-risk NBs (main exposure) and EBF discontinuation in the sixth month (outcome) were selected for adjustment (maternal education, maternal age, presence of gestational morbidity, smoking during pregnancy, parity, and number of prenatal consultations) (Fig 1) [40]. Based on the literature, these six variables were considered simultaneously associated with exposure and outcome.

The mothers were asked about smoking during pregnancy, being classified as “yes” or “no.” Passive smoking and smoke intensity were not assessed. Income and education were used as indicators of socioeconomic status. The mother’s education was classified as “up to elementary school” and “high school or above.” As for the income, the family’s monthly income was divided according to the value of the minimum wage (MW) in 2018 (approximately $576.00) as “up to two times the MW” and “equal to or greater than two times the MW.” Parity was defined as “primiparous” and “multiparous.” Gestational morbidity was classified as having at least one disease during pregnancy (“yes”) or none (“no”). The number of prenatal consultations was defined as “adequate” (six consultations or more) and “inadequate” (less than six consultations).

EBF time, in days, was calculated using the birth date and the evaluation dates (monthly until the sixth month of life). In case of follow-up discontinuation, EBF continuation, or death, discontinuation and last evaluation dates were used as censoring. Time was treated as a counting process.

The Kaplan-Meier method and the Log-rank test were used with the estimated median EBF duration to identify differences between survival curves stratified by each NB group. Subsequently, the Kaplan-Meier method was performed considering the time until EBF discontinuation stratified by the variable feeding practice at hospital discharge with a sample of NBs from the risk groups.

The semi-parametric Cox model was used to interpret the crude effects and, subsequently, a multiple model was used to obtain the adjusted effects, considering the minimum set of variables selected by the DAG. The effects were interpreted by the hazard ratio (HR) of the simple (crude HR) and multiple (adjusted HR) models and their respective 95% confidence intervals (CI 95%). There was no lack of proportionality in the Schoenfeld residuals, and no influential points were verified by the score residuals.

P-values <0.05 suggest significant differences. Due to the possible bias introduced by the exclusive use of p-values for decision making [41], CI 95% were provided for the point estimates of the study. The FactoMineR, cluster, factoextra, survival, survminer, and finalfit packages of the R software version 3.6.3 were used in the analysis [42–46].

### Ethics approval

The study was approved by the Research Ethics Committee of IFF/FIOCRUZ (protocol number: 1.930.996–2017). All procedures performed in studies involving human participants were in accordance with the ethical standards of the institutional and/or national research committee. Written informed consent was obtained from all mothers over 18 years old included in the study. A parent or guardian was on behalf of any participants under the age of 18 to write informed consent.

## Results

Table 1 shows the main characteristics of the study participants by outcome (Table 1). The risk groups (1 and 2) represent 47% of the cohort, 53% of NBs were full-term and healthy. Of these, some had a potential risk at birth due to the presence of at least one gestational morbidity. EBF prevalence was 65.2% at discharge (CI95% 62.2–68.3) and 9.3% at six months (CI95% 5.2–13.7). Of the total population, there was a loss of follow-up of 75 (7.5%) children.

**Table 1:** Characterization of the study population by feeding practice at hospital discharge and at six months of life, Rio de Janeiro, RJ, Brazil, 2018.

Characteristics	HOSPITAL DISCHARGE							SIXTH MONTH OF LIFE						
	Total			EBF		EBF discontinuation		Total			EBF		EBF discontinuation	
				(n = 641)		(n = 342)					(n = 147)		(n = 733)	
	n	%	CI95%	%	CI95%	%	CI95%	n	%	CI95%	%	CI95%	%	CI95%
**Neonatal grouping**^**a**^	983							880						
Full-term and healthy		53.0	(49.8–56.2)	65.4	(61.5–69.0)	29.8	(25.0–35.0)		54.3	(51.0–57.6)	76.9	(69.2–83.4)	49.8	(46.1–53.5)
Risk group 1		28.5	(25.7–31.4)	23.2	(20.0–26.7)	38.3	(33.1–43.7)		27.0	(24.1–30.1)	16.3	(10.7–23.3)	29.2	(25.9–32.6)
Risk group 2		18.5	(16.1–21.1)	11.4	(9.0–14.1)	31.9	(27.0–37.1)		18.6	(16.1–21.4)	6.8	(3.3–12.2)	21.0	(18.1–24.1)
**Twinning**	983							880						
No		85.1	(82.8–87.3)	89.5	(86.9–91.8)	76.9	(72.1–81.3)		84.0	(81.4–86.3)	95.9	(91.3–98.5)	81.6	(78.6–84.3)
Yes		14.9	(12.7–17.2)	10.5	(8.2–13.1)	23.1	(18.7–27.9)		16.0	(13.7–18.6)	4.1	(1.5–8.7)	18.4	(15.7–21.4)
**Gestational morbidity**	983							880						
No		51.8	(48.6–54.9)	54.4	(50.5–58.4)	46.8	(41.4–52.2)		52.2	(48.8–55.5)	55.1	(46.7–63.3)	51.6	(47.9–55.2)
Yes		48.2	(45.1–51.4)	45.6	(41.6–49.5)	53.2	(47.8–58.6)		47.8	(44.5–51.2)	44.9	(36.7–53.3)	48.4	(44.8–52.1)
**Perinatal morbidity**	983							880						
No		59.1	(56.0–62.2)	70.4	(66.7–73.9)	38.0	(32.8–43.4)		60.8	(57.5–64.0)	78.2	(70.7–84.6)	57.3	(53.6–60.9)
Yes		40.9	(37.8–44.0)	29.6	(26.1–33.3)	62.0	(56.6–67.2)		39.2	(36.0–42.5)	21.8	(15.4–29.3)	42.7	(39.1–46.4)
**Birth weight**	983							880						
< 1500g		3.2	(2.2–4.4)	0.6	(0.2–1.6)	7.9	(5.3–11.3)		2.8	(1.8–4.2)	0.0	(0.0–2.5)	3.4	(2.2–5.0)
1500–2500g		15.8	(13.5–18.2)	9.4	(7.2–11.9)	27.8	(23.1–32.8)		15.7	(13.3–18.3)	6.8	(3.3–12.2)	17.5	(14.8–20.4)
> 2500g		81.1	(78.5–83.5)	90.0	(87.4–92.2)	64.3	(59.0–69.4)		81.5	(78.7–84.0)	93.2	(87.8–96.7)	79.1	(76.0–82.0)
**Gestational age**	983							880						
≥ 37 weeks		78.4	(75.7–81.0)	86.6	(83.7–89.1)	63.2	(57.8–68.3)		79.3	(76.5–81.9)	90.5	(84.5–94.7)	77.1	(73.9–80.1)
< 37 weeks		21.6	(19.0–24.3)	13.4	(10.9–16.3)	36.8	(31.7–42.2)		20.7	(18.1–23.5)	9.5	(5.3–15.5)	22.9	(19.9–26.1)
**Type of delivery**	983							880						
C-section		58.3	(55.1–61.4)	50.4	(46.4–54.3)	73.1	(68.1–77.7)		59.0	(55.6–62.2)	57.1	(48.7–65.3)	59.3	(55.7–62.9)
Trans-pelvic		41.7	(38.6–44.9)	49.6	(45.7–53.6)	26.9	(22.3–31.9)		41.0	(37.8–44.4)	42.9	(34.7–51.3)	40.7	(37.1–44.3)
**Maternal education**	980							878						
Up to elementary education		38.2	(35.1–41.3)	37.3	(33.5–41.2)	39.8	(34.5–45.2)		36.4	(33.3–39.7)	30.6	(23.3–38.7)	37.6	(34.1–41.2)
High school or above		61.8	(58.7–64.9)	62.7	(58.8–66.5)	60.2	(54.8–65.5)		63.6	(60.3–66.7)	69.4	(61.3–76.7)	62.4	(58.8–65.9)
**Family income**^**b**^	806							738						
≥ 2 times the minimum wage ($576.00)		60.7	(57.2–64.1)	62.2	(57.9–66.3)	57.8	(51.7–63.6)		61.5	(57.9–65.0)	66.4	(57.2–74.8)	60.6	(56.6–64.5)
< 2 times the minimum wage ($576.00)		39.3	(35.9–42.8)	37.8	(33.7–42.1)	42.2	(36.4–48.3)		38.5	(35.0–42.1)	33.6	(25.2–42.8)	39.4	(35.5–43.4)
**Maternal age**	980							878						
< 20 years old		17.4	(15.1–20.0)	16.9	(14.1–20.1)	18.4	(14.5–22.9)		12.5	(10.4–14.9)	15.6	(10.2–22.5)	11.9	(9.6–14.5)
20 to 34 years old		68.6	(65.6–71.5)	68.7	(64.9–72.2)	68.4	(63.2–73.3)		69.5	(66.3–72.5)	70.1	(62.0–77.3)	69.4	(65.9–72.7)
≥ 35 years old		14.0	(11.9–16.3)	14.4	(11.8–17.4)	13.2	(9.8–17.2)		18.0	(15.5–20.7)	14.3	(9.1–21.0)	18.7	(16.0–21.8)
**Smoking during pregnancy**	976							874						
No		91.8	(89.9–93.4)	92.1	(89.7–94.1)	91.2	(87.7–94.0)		92.4	(90.5–94.1)	93.2	(87.8–96.7)	92.3	(90.1–94.1)
Yes		8.2	(6.6–10.1)	7.9	(5.9–10.3)	8.8	(6.0–12.3)		7.6	(5.9–9.5)	6.8	(3.3–12.2)	7.7	(5.9–9.9)
**Parity and previous breastfeeding experience**	957							857						
Multiparous with prior BF		44.4	(41.2–47.6)	44.8	(40.9–48.8)	43.7	(38.3–49.2)		44.5	(41.1–47.9)	43.4	(35.2–51.9)	44.7	(41.0–48.4)
Multiparous with no prior BF		50.6	(47.4–53.8)	50.7	(46.7–54.7)	50.3	(44.8–55.8)		5.4	(4.0–7.1)	2.8	(0.8–6.9)	5.9	(4.3–7.9)
Primiparous		5.0	(3.7–6.6)	4.5	(3.0–6.4)	6.0	(3.7–9.2)		50.2	(46.8–53.6)	53.8	(45.3–62.1)	49.4	(45.7–53.2)
**Number of prenatal consultations**	979							877						
Adequate		89.7	(87.6–91.5)	93.2	(91.0–95.1)	83.0	(78.6–86.9)		90.6	(88.5–92.5)	90.5	(84.5–94.7)	90.7	(88.3–92.7)
Inadequate		10.3	(8.5–12.4)	6.8	(4.9–9.0)	17.0	(13.1–21.4)		9.4	(7.5–11.5)	9.5	(5.3–15.5)	9.3	(7.3–11.7)
**Place of hospitalization**	982							879						
Rooming-in		69.8	(66.8–72.6)	82.4	(79.2–85.2)	46.0	(40.7–51.5)		71.9	(68.8–74.9)	87.7	(81.2–92.5)	68.8	(65.3–72.1)
Neonatal intensive care unit		30.2	(27.4–33.2)	17.6	(14.8–20.8)	54.0	(48.5–59.3)		28.1	(25.1–31.2)	12.3	(7.5–18.8)	31.2	(27.9–34.7)
**Work and maternity leave**	965							867						
Does not work		55.4	(52.2–58.6)	53.6	(49.6–57.5)	58.9	(53.4–64.2)		54.6	(51.2–57.9)	57.6	(49.1–65.8)	53.9	(50.2–57.6)
Works, with 4-month ML		4.4	(3.2–5.8)	4.1	(2.7–6.0)	4.7	(2.7–7.6)		4.5	(3.2–6.1)	5.6	(2.4–10.7)	4.3	(2.9–6.0)
Works, with 6-month ML		4.0	(2.9–5.5)	4.6	(3.1–6.6)	3.0	(1.4–5.4)		4.3	(3.0–5.8)	3.5	(1.1–7.9)	4.4	(3.0–6.2)
Works at home		25.0	(22.3–27.8)	26.5	(23.1–30.1)	22.2	(17.9–27.0)		25.5	(22.6–28.5)	22.2	(15.7–29.9)	26.1	(23.0–29.5)
Works, without ML		11.2	(9.3–13.4)	11.2	(8.8–13.9)	11.2	(8.1–15.1)		11.2	(9.2–13.5)	11.1	(6.5–17.4)	11.2	(9.0–13.7)
**Skin to skin contact in the delivery room**	977							874						
No		52.5	(49.3–55.7)	42.9	(39.0–46.8)	70.6	(65.4–75.4)		51.9	(48.6–55.3)	46.6	(38.3–55.0)	53.0	(49.3–56.7)
Yes		47.5	(44.3–50.7)	57.1	(53.2–61.0)	29.4	(24.6–34.6)		48.1	(44.7–51.4)	53.4	(45.0–61.7)	47.0	(43.3–50.7)
**Use of pacifier during hospitalization**	980							876						
No		85.7	(83.4–87.8)	94.7	(92.7–96.3)	68.8	(63.6–73.7)		86.6	(84.2–88.8)	96.6	(92.1–98.9)	84.7	(81.9–87.2)
Yes		14.3	(12.2–16.6)	5.3	(3.7–7.3)	31.2	(26.3–36.4)		13.4	(11.2–15.8)	3.4	(1.1–7.9)	15.3	(12.8–18.1)

Note

^a^grouping: group of healthy full-term newborns; risk group 1—characterized by newborns with surgical morbidities and genetic syndrome; risk group 2—characterized by preterm newborns and low birth weight.

^b^minimum wage 2016 - [http://www.planalto.gov.br/ccivil_03/_Ato2015-2018/2016/Decreto/D8948.htm]; [http://receita.economia.gov.br/orientacao/tributaria/declaracoes-e-demonstrativos/ecf-escrituracao-contabil-fiscal/taxas-de-cambio-incluindo-valor-do-dolar-para-fins-fiscais-irpj-AC-anteriores]. HR = hazard ratio; CI = confidence interval; BF = breastfeeding; EBF = exclusive breastfeeding; ML = maternity leave.

The study included 1,003 participants. MCA with the ten risk variables revealed that six presented no relative contributions to inertia. The MCA of the four remaining variables (birth weight, gestational age, perinatal morbidity, and twinning) identified three NB groups with 68.9% of adjusted inertia (Fig 1).

Group 1 (called healthy full-term group) was characterized by NBs from single pregnancies, healthy, full-term, with good birth weight. Group 2 (called risk group 1) was characterized by NBs with perinatal morbidity, with most surgical pathologies and genetic syndromes being concentrated in this group (some with extreme and very low weight), and mostly from single pregnancies (only 12.3% twins). Group 3 (called risk group 2) included most of the NBs with very low birth weight, preterm, and with perinatal morbidities. Most twins were also in this group. Few surgical malformations and genetic syndromes were allocated to group 3 (of these, 94% were underweight and some were preterm) (Fig 1).

Although supplementary variables are not part of the group construction, there was a relationship between active and supplementary variables in such a way that the social and biological protective categories are close to the group of healthy full-term NB, while low maternal education, gestational morbidity, surgical morbidity, genetic syndrome, and Apgar below 7 in the fifth minute are on the positive side of dimension 1, where the two risk groups are located (Fig 1).

Cluster analysis confirmed the three groups found in MCA and classified the subjects in an outcome variable with three risk categories. The association of maternal and child characteristics according to the three identified groups are presented in S2 File. In general, all the biological risk criteria presented a greater proportion in groups 2 and 3 (risk group 1 and risk group 2) than in group 1 (healthy full-term). In all groups, the prevalence of pregnancy morbidity varied between 41% and 56%, and social characteristics did not differ between groups.

Table 2 shows the prevalence of each feeding practice by NB group. The group of healthy NBs received EBF in the sixth month approximately two and four times higher than in risk groups 1 and 2, respectively. Most NBs in all groups were being breastfed in the sixth month of life (with infant formula supplementation).

**Table 2:** Prevalence of breastfeeding at six months of life by risk neonatal profile in a referral center for high-risk infants, Rio de Janeiro, RJ, Brazil, 2018.

Neonatal grouping^a^	Total		EBF		PB		PBF		NBF	
	n (%)	CI 95%	n (%)	CI 95%	n (%)	CI 95%	n (%)	CI 95%	n (%)	CI 95%
Full-term and healthy	478 (54)	(50.9–57.6)	113 (23.6)	(19.8–27.7)	107 (22.4)	(18.7–26.3)	237 (49.6)	(45.0–54.1)	21 (4.4)	(2.7–6.6)
Risk group 1	238 (27)	(24.1–30.1)	24 (10)	(6.5–14.6)	34 (14.3)	(10.1–19.3)	142 (59.7)	(53.1–65.9)	38 (16)	(11.5–21.2)
Risk group 2	164 (19)	(16.1–21.3)	10 (6.1)	(2.9–10.9)	9 (5.5)	(2.5–10.1)	117 (71.3)	(63.7–78.1)	28 (17.1)	(11.6–23.7)

Note

^a^grouping: group of healthy full-term newborns; risk group 1—characterized by newborns with surgical morbidities and genetic syndrome; risk group 2—characterized by preterm newborns and low birth weight. EBF = exclusive breastfeeding; PB = predominant breastfeeding; PBF = partial breastfeeding; NBF = non-breastfed; CI = confidence interval.

**Fig 2:**
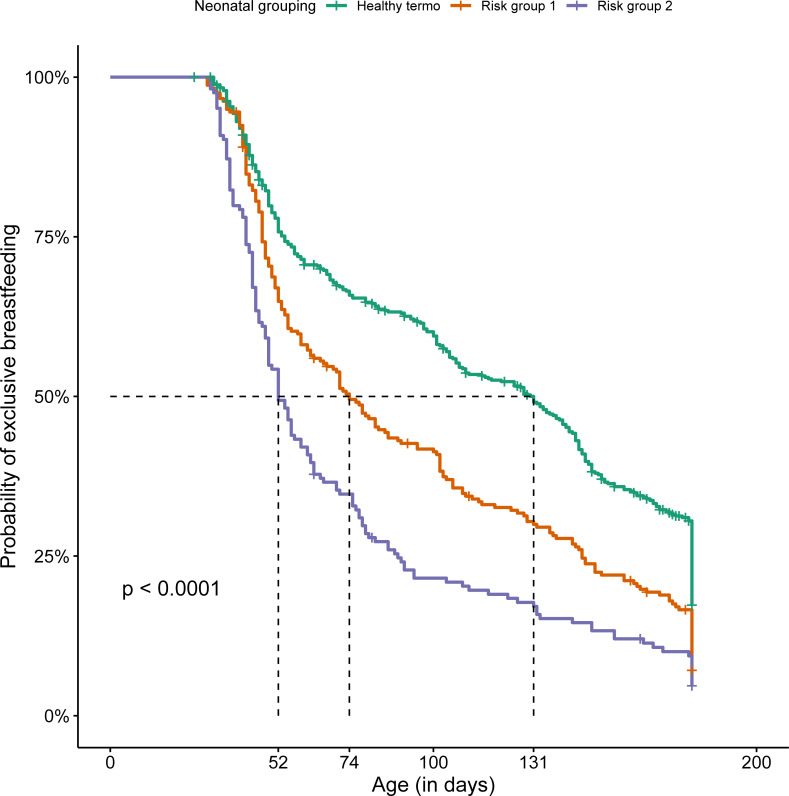
Kaplan-Meier of the exclusive breastfeeding time of 880 newborns from a high-risk fetal, neonatal, and infant center by groups, Brazil, 2018. Note: Risk group 1—characterized by newborns with surgical morbidities and genetic syndrome; risk group 2—characterized by preterm newborns and low birth weight. Log-rank < 0.001 EBF = exclusive breastfeeding; PBF = partial breastfeeding; BF = bottle-feeding.

The median duration of EBF was 91 days, being longer for the group composed mostly of healthy full-term NBs (131 days) than for risk groups 1 and 2 (74 and 52 days, respectively) (Fig 2).

**Fig 3:**
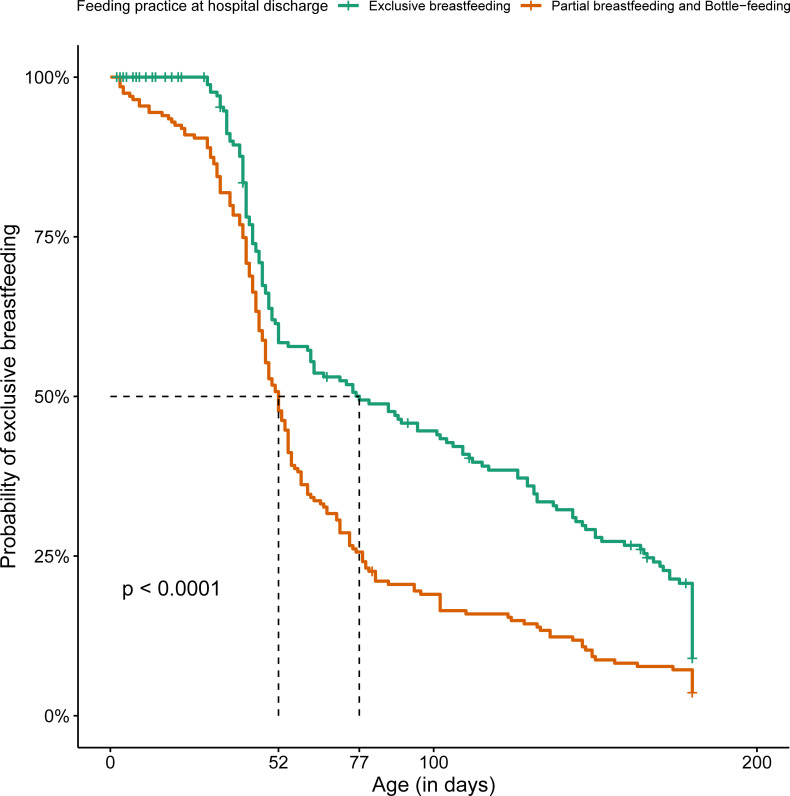
Kaplan-Meier of exclusive breastfeeding according to the feeding practice at hospital discharge of the 393 newborns in risk groups 1 and 2, Brazil, 2018.

Fig 3 shows the difference between survival curves by feeding practice at hospital discharge in risk groups 1 and 2. NBs at risk who received formulas at hospital discharge had a shorter median EBF duration.

The distribution of main independent variables and HR for each NB group is shown in Table 3. The multiple model shows a gradient effect that increases in the risk groups when adjusted by the minimum set of DAG variables, with NBs in risk groups 1 (surgical procedure and genetic syndrome) and 2 (preterm babies, low birth weight, and twins) having, respectively, 40% and 111% more risk of EBF discontinuation compared to the group of healthy full-term NBs.

**Table 3:** Single and multi-covariate Cox regression models of exclusive breastfeeding discontinuation by risk neonatal profile in a referral center for high-risk infants, Rio de Janeiro, RJ, Brazil, 2018.

Neonatal grouping^a^	crude HR	adjusted HR
	(CI 95%)	(CI 95%)
Full-term and healthy	-	-
Risk group 1	1.41 (1.17–1.71)	1.40 (1.15–1.69)
Risk group 2	2.14 (1.73–2.65)	2.11 (1.69–2.62)

Note

^a^grouping: group of healthy full-term newborns; risk group 1—characterized by newborns with surgical morbidities and genetic syndrome; risk group 2—characterized by preterm newborns and low birth weight. Multiple model adjusted by DAG: maternal education, maternal age, gestational morbidity, smoking during pregnancy, parity and number of prenatal consultations; n = 880.

HR = hazard ratio; CI = confidence interval.

## Discussion

In this study, correspondence and cluster analyses identified three groups of NBs with different biological patterns, including healthy full-term NBs and the two risk groups. The prevalence and median duration of EBF decreased proportionally in all groups, despite the risk. After adjusting for potential confounders, an increased gradient effect was identified for EBF discontinuation in groups of NBs at risk. In addition, it was found that EBF during hospitalization predicts a longer median duration of this practice in risk groups.

Weight and prematurity were important determinants between the two risk groups. Weight is an important indicator in the prenatal period and throughout the child’s follow-up [47]. In this study, in addition to being an indicator of growth and risk, it also had a major role in defining risk groups. This characteristic is recognized by defining and directing public strategies and policies, as well as for being a proxy for a set of components such as nutrition, organic structure, short and long term consequences, and mortality [22].

The socioeconomic position of the family must be distinguished in the classification of biological risk for NBs in future studies with other populations. Social risk information provides a dimension of the social context in which the children will develop and their health conditions. Although the presence of gestational morbidity was not considered relevant for defining the groups by correspondence analysis, this characteristic was associated mainly between groups with and without risk, representing a potential risk at birth [48, 49]. Twinning was not associated with actual risk conditions, but with the potential risk of prematurity and low weight, as previously reported [49, 50].

As expected, there is a proportional decrease in the prevalence of EBF between groups. The EBF rate in the sixth month of life for NBs undergoing surgical procedures was similar to the rate found in a previous study in Mexico with the same population [26]. Preterm NBs presented a lower prevalence of EBF compared to a cohort in Denmark [51], and slightly higher compared to another Brazilian study conducted at the same institute [52]. Even so, the prevalence of EBF is below the recommended range [53].

A cross-sectional study on the situation of BF in a healthy population in Brazil found a median EBF duration of 54.1 days in children under six months [28]. In the present study, the median duration was longer in the group composed mostly of healthy full-term NBs and risk group 1 (131 and 74 days, respectively), with a similar duration between risk group 2 (52 days) and the result found in a previous Brazilian study with healthy children. Contrary to what was expected, there was a longer duration of EBF even in the context of risk, which represents an important advance in this indicator.

Despite this progress, the difference between groups regarding median EBF duration, which is lower in the presence of risk at birth, is worrisome. Such a result can be attributed to long hospital stay, resulting in greater exposure to conditions that can inhibit EBF, and to the feeding practice during hospital stay, which would indicate the influence of health practices and services [54]. Similarly to previous studies, the use of infant formula during hospitalization resulted in shorter EBF, which provides greater protection and health recovery [55–57], while EBF during hospitalization predicts longer EBF duration [54, 58]. McCoy & Heggie [55] describe a number of factors related to unnecessary supplementation and offer consistent reasons to avoid the frequent use of formulas, a practice that can affect BF time and practices. Possible reasons for the different prevalences and durations of EBF in risk groups 1 and 2 are due to the characteristics of the groups’ formation (clinical complexity), which result in different lengths of hospitalization and provided care, therefore the challenge to promote EBF among different risk groups remains.

Preterm births, complications during delivery (neonatal asphyxia), infections, and congenital anomalies are the main causes of neonatal deaths in 2018 [59]. Many deaths could be prevented or treated with simple interventions, as long as they are accessible. BF is a simple and low-cost intervention with positive results in neonatal survival and injury prevention in survivors [10].

Considering that most children in all groups were being breastfed and supplemented in the sixth month of life, it is worth highlighting the potential for increasing the practice of EBF. This condition requires researchers and health care providers to reflect and change. NBs at risk need extra support and intensified efforts [21].

Policies and guidelines promoting BF vary worldwide, and there is a lack of standardization regarding how policies and practices are implemented in the context of high risk [23, 60, 61]. To date, there is no specific policy or program in Brazil to promote and support all the particularities of an NB hospitalized in a neonatal unit that increase BF rates.

Of the Brazilian BF incentive programs, the presence of a HMB in the hospital has a consistent impact on BF rates and on the use of human milk in the neonatal intensive care unit (NICU). This impact shows the need to strengthen HMB as reference BF centers, especially for the population at risk. In Brazil, Human Milk Banks collect and distribute certified milk for high-risk NBs, and also offer breastfeeding support, especially regarding the mother-hospitalized-high-risk-infant dyad. All assistance and follow-up are carried out by the team of nurses and pediatricians of the HMB, with greater expertise and ability to clinically manage breastfeeding with this group of high-risk NBs, offering care and defining specific procedures during the prenatal period, all the hospitalization, and the first follow-up visits after hospital discharge [58]. A study comparing the rate of EBF in 4,277 babies with very low birth weight hospitalized in 83 NICUs in Italy reported higher EBF rates at hospital discharge in NICU that had an HMB in the hospital (29.6%) compared to NICU with no HMB (16.0%) [62]. A study by Parker et al. (2016) showed that NBs discharged from the NICU two years after implantation of the HMB were six times more likely to receive breast milk at hospital discharge compared to those discharged before the program was implemented. The HMB collects, processes, and distributed pasteurized human milk, strengthening the BF culture and the use of human milk in the NICU. Furthermore, this service has great impact in promoting this practice in high complexity services [63, 64].

The present study shows that the main exposure (risk at birth) represents a construct that captures dimensions beyond severity that involve healthcare quality and hospital practices. Thus, further studies should investigate the effect of these determinants as the main exposure for causal inference.

A number of limitations have to be mentioned. The first one is the lack of generality to other populations. As for the survival analysis, the lack of an exact EBF discontinuation date was circumvented by the use of the counting process (although there is inaccuracy in the day, there was no inaccuracy in the month, as a monthly call was made). Another limitation refers to the periodicity of the evaluations; however, the study used mixed logistic models, with no significantly different results and with the disadvantage of excluding NBs with only a monthly evaluation and effect overestimation. Another approach, such as the assessment of changed BF types over time, was verified by multi-state models, but the low frequency of changes allowed no adequate adjustment, which justified the approach used in this article. Other limitations refer to the correspondence analysis technique, which has an exploratory character, because this population comes from referral center whose results can be only extrapolated to similar populations.

This is the first study conducted in Brazil with different and representative categories of NBs at risk, with strengths such as (i) the longitudinal design of the NB cohort from a referral center for high risk followed up until six months of life; (ii) population size with great risk variability; (iii) high adherence to follow-up (follow-up loss of 7.5%); and (iv) a data control and quality assurance process. The loss of follow-up was assessed in a previous study conducted by the same group, which identified no differences between lost and remaining participants [31].

The identification of different risk patterns in this study contributes to the formulation of strategies and public policies. The planning of these strategies can start with the different types of support which show beneficial effects on longer BF duration in the risk infants, as suggested by previous studies, such as use of prenatal consultation, supplementation methods during the transition to breastfeeding (cup feeding and supplemental nursing system device), avoidance of use of pacifiers and bottle feeding, skilled support from trained staff and others [23, 65, 66]. The use of multivariate statistics combined with dimensionality reduction analysis techniques (correspondence and clusters) proved to be useful to describe risk groups in NBs monitored at a national referral center for high fetal, neonatal, and infant risk. Cluster analysis results can guide health teams and managers in planning and establishing educational campaigns, preventive actions, and early interventions such as BF promotion and support for populations at risk.

Finally, this study indicates the need to strengthen the BF culture in high complexity neonatal units and offers robust arguments to equitably preserve EBF during hospitalization for this at-risk population.

## Conclusion

This study illustrates the use of dimensionality reduction analyses (correspondence and clusters) in a large cohort of NBs in a referral center for high fetal, neonatal, and infant risk. The analysis identified three groups with different biological risk patterns, including healthy full-term NBs and two groups at different risk levels. The study additionally describes the relationship between characteristics related to social and potential risk and the identified groups. In this sense, this study can improve clinical practice conducts in the development of efficient strategies and future research.

The prevalence and median duration of EBF proportionally decreased in the group of healthy full-term NBs and risk groups 1 and 2. EBF during hospitalization predicts a longer median duration of this practice in risk groups. The risk of EBF discontinuation was higher in the risk groups, with a gradual effect even when adjusted by several factors. However, most children in all groups continued BF with supplementation in the sixth month of life, which indicates the potential for increasing EBF.

Effective and targeted interventions to promote, protect, and support EBF in high-risk populations need to be implemented, strengthened, expanded, and associated with other interventions at different levels, so that EBF practices increase, and all their determinants are responsive. In this sense, accessible and good quality health services should recognize situations of greater vulnerability to low BF rates and discontinuation and implement measures to reduce them.

## Supporting information

S1 File DAG of the plan to analyze the association between high-risk NBs and EBF at sixth month of life. Note: EBF = exclusive breastfeeding; BF = breastfeeding; PDHM = pasteurized donor human milk; HMB = Human Milk Bank; NPO = nothing through the mouth. Green node (high-risk newborn) = exposure variable; blue node with “I” (EBF at sixth month of life) = variable outcome; empty blue node = mediating variables; red node = explanatory variables (confounding).
(TIF)

S2 File Group characterization by biological risk, potential risk, and social risk of a cohort of newborns, Rio de Janeiro, RJ, Brazil, 2018. Note: * test could not be performed due to 0 count. CI = confidence interval. P-value <0.05 based on the Pearson’s chi-square /Fisher’s exact tests.
(DOCX)

## Abbreviations

BF: Breastfeeding
DAG: Directed Acyclic Graph
EBF: Exclusive Breastfeeding
HR: Hazard ratio
PB: Predominant Breastfeeding
PBF: Partial Breastfeeding
FIOCRUZ: Oswaldo Cruz Foundation
HMB: Human Milk Bank
IFF: National Institute of Women, Children and Adolescents Health Fernandes Figueira
MCA: Multiple correspondence analysis
NB: Newborn
NBF: Non-Breastfed
NICU: Neonatal Intensive Care Unit
