# Supplementary figures and images for: The effect of risk at birth on breastfeeding duration and exclusivity: A cohort study at a Brazilian referral center for high-risk neonates and infants
Source: PLoS One. 2021 Aug 6;16(8):e0255190. doi: 10.1371/journal.pone.0255190 (PMC8346259; doi:10.1371/journal.pone.0255190)

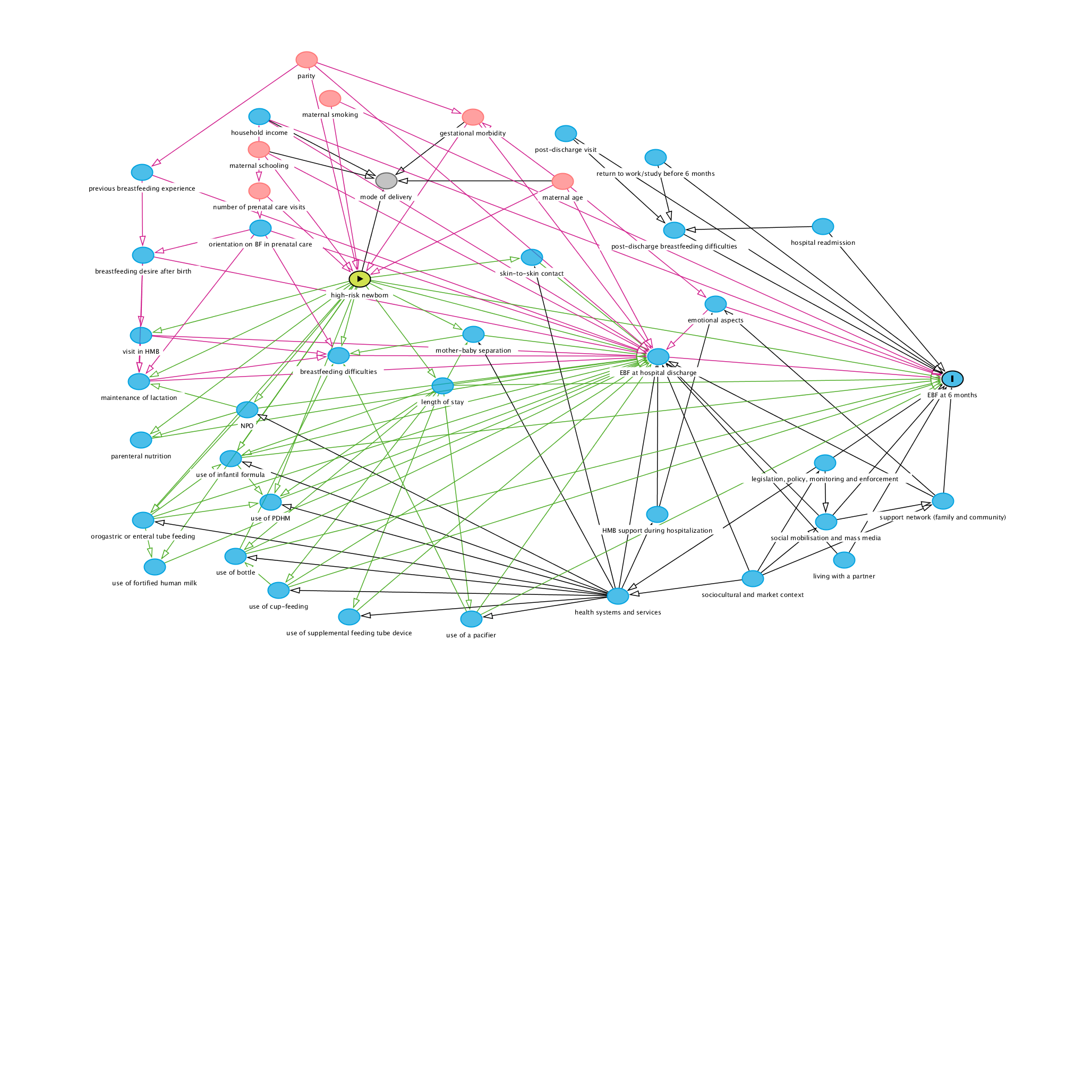

Supplement: S1 File — Note: EBF = exclusive breastfeeding; BF = breastfeeding; PDHM = pasteurized donor human milk; HMB = Human Milk Bank; NPO = nothing through the mouth. Green node (high-risk newborn) = exposure variable; blue node with “I” (EBF at sixth month of life) = variable outcome; empty blue node = mediating variables; red node = explanatory variables (confounding). (TIF) [file pone.0255190.s001.tif]
