# Supplementary material for: The effect of risk at birth on breastfeeding duration and exclusivity: A cohort study at a Brazilian referral center for high-risk neonates and infants
Source: PLoS One. 2021 Aug 6;16(8):e0255190. doi: 10.1371/journal.pone.0255190 (PMC8346259; doi:10.1371/journal.pone.0255190)
Supplement: S2 File — Note: * test could not be performed due to 0 count. CI = confidence interval. P-value <0.05 based on the Pearson’s chi-square /Fisher’s exact tests. (DOCX) [file pone.0255190.s002.docx]

| \| S2 File – Group characterization by biological risk, potential risk, and social risk of a cohort of newborns, Rio de Janeiro, RJ, Brazil, 2018. \| \| \| \| \| \| \| \| \| \| --- \| --- \| --- \| --- \| --- \| --- \| --- \| --- \| --- \| \|  \| Total \| **Group of healthy full-term**  **newborns** \| \| **Risk group 1** \| \| **Risk group 2** \| \| p-value \| \|  \| n (%) \| n (%) \| CI95% \| n (%) \| CI 95% \| n (%) \| CI 95% \| \| **BIOLOGICAL RISK** \|  \|  \|  \|  \|  \|  \|  \|  \| \| ***Perinatal morbidity*** \|  \|  \|  \|  \|  \|  \|  \| -* \| \| No \| 581 (57.9) \| 521 (100.0) \| (99.3-100.0) \| 49 (16.7) \| (12.6-21.5) \| 11 (5.8) \| (2.9-10.2) \|  \| \| Yes \| 422 (42.1) \| 0 (0.0) \| (0.0-0.7) \| 244 (83.3) \| (78.5-87.4) \| 178 (94.2) \| (89.8-97.1) \|  \| \| ***Congenital malformation*** \|  \|  \|  \|  \|  \|  \|  \| -* \| \| No \| 873 (87.0) \| 521 (100.0) \| (99.3-100.0) \| 194 (66.2) \| (60.5-71.6) \| 158 (83.6) \| (77.5-88.6) \|  \| \| Yes \| 130 (13.0) \| 0 (0.0) \| (0.0-0.7) \| 99 (33.8) \| (28.4-39.5) \| 31 (16.4) \| (11.4-22.5) \|  \| \| ***Genetic syndrome*** \|  \|  \|  \|  \|  \|  \|  \| -* \| \| No \| 992 (98.9) \| 521 (100.0) \| (99.3-100.0) \| 286 (97.6) \| (95.1-99.0) \| 185 (97.9) \| (94.7-99.4) \|  \| \| Yes \| 11 (1.1) \| 0 (0.0) \| (0.0-0.7) \| 7 (2.4) \| (1.0-4.9) \| 4 (2.1) \| (0.6-5.3) \|  \| \| ***Birth weight*** \|  \|  \|  \|  \|  \|  \|  \| -* \| \| < 1,500 g \| 38 (3.8) \| 0 (0.0) \| (0.0-0.7) \| 20 (6.8) \| (4.2-10.3) \| 18 (9.5) \| (5.7-14.6) \|  \| \| 1,500–2,499g \| 159 (15.9) \| 0 (0.0) \| (0.0-0.7) \| 14 (4.8) \| (2.6-7.9) \| 145 (76.7) \| (70.0-82.5) \|  \| \| >2,500 g \| 806 (80.4) \| 521 (100.0) \| (99.3-100.0) \| 259 (88.4) \| (84.2-91.8) \| 26 (13.8) \| (9.2-19.5) \|  \| \| ***Gestational age*** \|  \|  \|  \|  \|  \|  \|  \| -* \| \| Full-term \| 779 (77.7) \| 521 (100.0) \| (99.3-100.0) \| 222 (75.8) \| (70.4-80.6) \| 36 (19.0) \| (13.7-25.4) \|  \| \| Preterm \| 224 (22.3) \| 0 (0.0) \| (0.0-0.7) \| 71 (24.2) \| (19.4-29.6) \| 153 (81.0) \| (74.6-86.3) \|  \| \| ***Fifth minute Apgar score*** \|  \|  \|  \|  \|  \|  \|  \| 0.092 \| \| < 7 \| 35 (3.5) \| 12 (2.3) \| (1.2-4.0) \| 13 (4.4) \| (2.4-7.5) \| 10 (5.3) \| (2.6-9.5) \|  \| \| > 7 \| 968 (96.5) \| 509 (97.7) \| (96.0-98.8) \| 280 (95.6) \| (92.5-97.6) \| 179 (94.7) \| (90.5-97.4) \|  \| \| **POTENTIAL RISK** \|  \|  \|  \|  \|  \|  \|  \|  \| \| ***Gestational morbidity*** \|  \|  \|  \|  \|  \|  \|  \| < 0.001 \| \| No \| 518 (51.6) \| 305 (58.5) \| (54.2-62.8) \| 128 (43.7) \| (37.9-49.6) \| 85 (45.0) \| (37.7-52.4) \|  \| \| Yes \| 485 (48.4) \| 216 (41.5) \| (37.2-45.8) \| 165 (56.3) \| (50.4-62.1) \| 104 (55.0) \| (47.6-62.3) \|  \| \| ***Twinning*** \|  \|  \|  \|  \|  \|  \|  \| -* \| \| No \| 854 (85.1) \| 521 (100.0) \| (99.3-100.0) \| 257 (87.7) \| (83.4-91.2) \| 76 (40.2) \| (33.2-47.6) \|  \| \| Yes \| 149 (14.9) \| 0 (0.0) \| (0.0-0.7) \| 36 (12.3) \| (8.8-16.6) \| 113 (59.8) \| (52.4-66.8) \|  \| \| **SOCIAL RISK** \|  \|  \|  \|  \|  \|  \|  \|  \| \| ***Maternal education*** \|  \|  \|  \|  \|  \|  \|  \| 0.100 \| \| Up to elementary education \| 383 (38.3) \| 188 (36.2) \| (32.0-40.4) \| 110 (37.8) \| (32.2-43.6) \| 85 (45.0) \| (37.7-52.4) \|  \| \| High school or above \| 617 (61.7) \| 332 (63.8) \| (59.6-68.0) \| 181 (62.2) \| (56.4-67.8) \| 104 (55.0) \| (47.6-62.3) \|  \| \| ***Family income*** \|  \|  \|  \|  \|  \|  \|  \| 0.470 \| \| > 2 times the minimum wages \| 498 (60.5) \| 267 (64.0) \| (59.2-68.6) \| 138 (55.0) \| (48.6-61.2) \| 93 (60.0) \| (51.8-67.8) \|  \| \| < 2 times the minimum wages \| 325 (39.5) \| 150 (36.0) \| (31.4-40.8) \| 113 (45.0) \| (38.8-51.4) \| 62 (40.0) \| (32.2-48.2) \|  \| \| ***Maternal age*** \|  \|  \|  \|  \|  \|  \|  \| 0.068 \| \| Up to 20 years old \| 139 (13.9) \| 81 (15.6) \| (12.6-19.0) \| 33 (11.3) \| (7.9-15.6) \| 25 (13.2) \| (8.7-18.9) \|  \| \| 20 to 34 years old \| 687 (68.7) \| 346 (66.5) \| (62.3-70.6) \| 207 (71.1) \| (65.6-76.3) \| 134 (70.9) \| (63.9-77.3) \|  \| \| 35 years old or more \| 174 (17.4) \| 93 (17.9) \| (14.7-21.5) \| 51 (17.5) \| (13.3-22.4) \| 30 (15.9) \| (11.0-21.9) \|  \|   Note: * test could not be performed due to 0 count.  CI = confidence interval. P-value <0.05 based on the Pearson’s chi-square /Fisher’s exact tests. |
| --- | --- | --- | --- | --- | --- | --- | --- | --- | --- | --- | --- | --- | --- | --- | --- | --- | --- | --- | --- | --- | --- | --- | --- | --- | --- | --- | --- | --- | --- | --- | --- | --- | --- | --- | --- | --- | --- | --- | --- | --- | --- | --- | --- | --- | --- | --- | --- | --- | --- | --- | --- | --- | --- | --- | --- | --- | --- | --- | --- | --- | --- | --- | --- | --- | --- | --- | --- | --- | --- | --- | --- | --- | --- | --- | --- | --- | --- | --- | --- | --- | --- | --- | --- | --- | --- | --- | --- | --- | --- | --- | --- | --- | --- | --- | --- | --- | --- | --- | --- | --- | --- | --- | --- | --- | --- | --- | --- | --- | --- | --- | --- | --- | --- | --- | --- | --- | --- | --- | --- | --- | --- | --- | --- | --- | --- | --- | --- | --- | --- | --- | --- | --- | --- | --- | --- | --- | --- | --- | --- | --- | --- | --- | --- | --- | --- | --- | --- | --- | --- | --- | --- | --- | --- | --- | --- | --- | --- | --- | --- | --- | --- | --- | --- | --- | --- | --- | --- | --- | --- | --- | --- | --- | --- | --- | --- | --- | --- | --- | --- | --- | --- | --- | --- | --- | --- | --- | --- | --- | --- | --- | --- | --- | --- | --- | --- | --- | --- | --- | --- | --- | --- | --- | --- | --- | --- | --- | --- | --- | --- | --- | --- | --- | --- | --- | --- | --- | --- | --- | --- | --- | --- | --- | --- | --- | --- | --- | --- | --- | --- | --- | --- | --- | --- | --- | --- | --- | --- | --- | --- | --- | --- | --- | --- | --- | --- | --- | --- | --- | --- | --- | --- | --- | --- | --- | --- | --- | --- | --- | --- | --- | --- | --- | --- | --- | --- | --- | --- | --- | --- | --- | --- | --- | --- | --- | --- | --- | --- | --- | --- | --- | --- | --- | --- | --- | --- | --- | --- | --- | --- | --- | --- | --- | --- | --- | --- | --- | --- | --- | --- | --- | --- | --- | --- | --- | --- | --- | --- | --- | --- | --- | --- | --- | --- | --- | --- | --- | --- | --- | --- | --- | --- | --- | --- | --- | --- | --- | --- | --- | --- | --- | --- | --- | --- | --- | --- | --- | --- | --- | --- | --- | --- | --- | --- | --- | --- | --- | --- | --- | --- | --- | --- | --- | --- | --- | --- | --- | --- | --- | --- | --- | --- | --- | --- | --- | --- | --- | --- | --- |
